# Overcontrol in anorexia nervosa: Elevated prefrontal activity and amygdala connectivity in a working memory task with food distractors

**DOI:** 10.1016/j.ijchp.2025.100544

**Published:** 2025-01-19

**Authors:** Sophie Pauligk, Maria Seidel, Franziska Ritschel, Daniel Geisler, Arne Doose, Ilka Boehm, Inger Hellerhoff, Franziska Ludwicki, Veit Roessner, Joseph A. King, Stefan Ehrlich

**Affiliations:** a Division of Psychological and Social Medicine and Developmental Neuroscience, Translational Developmental Neuroscience Section, Faculty of Medicine, Technische Universität Dresden, Fetscherstraße 74, 01307, Dresden, Germany; b Chair of Clinical Child and Adolescent Psychology, Faculty of Psychology, Technische Universität Dresden, Chemnitzer Straße 46a, 01187, Dresden, Germany; c Eating Disorder Research and Treatment Center, Department of Child and Adolescent Psychiatry, Faculty of Medicine, Technische Universität Dresden, Fetscherstraße 74, 01307, Dresden, Germany; d Department of Psychotherapy and Psychosomatic Medicine, Faculty of Medicine, Technische Universität Dresden, Fetscherstraße 74, 01307, Dresden, Germany

**Keywords:** Amygdala, Anorexia nervosa, Control, Emotion regulation, dlPFC, fMRI, Functional connectivity

## Abstract

Individuals with anorexia nervosa (AN) are thought to engage in excessive amounts of self-control, which may contribute to disorder development and maintenance. This “overcontrol” may explain previous findings of increased activity and connectivity in frontal brain regions involved in top-down control functions in response to diverse stimuli including emotionally salient visual food stimuli. However, these observations were made largely in tasks demanding explicit stimulus processing. Given the omnipresence of food cues and their particular relevance for AN, it deems important to test if these alterations are also present when food stimuli are task-irrelevant. To this end, we acquired functional magnetic resonance imaging data during a working memory 2-back task with images of high-caloric food as distractors in 32 acutely ill young women with AN and 32 age-matched female healthy control participants. Neural activity and connectivity was analyzed in *a priori* specified regions of interest involved in top-down control (dorsolateral prefrontal cortex; dlPFC) and affective processing (amygdala). Despite no group differences in task performance, activity of the left dlPFC was higher in AN compared with healthy controls across both food and non-food conditions. AN also showed increased negative connectivity between the left dlPFC and bilateral amygdalae. Generally increased dlPFC activation and altered dlPFC-amygdala connectivity in the context of our task is suggestive of excessive top-down control in AN. This activation pattern may reflect a neural substrate of overcontrol which occurs independent of external stimuli. This mechanism may be a potential treatment target, as it mirrors the clinical presentation of the disorder.

## Background

Anorexia nervosa is a serious eating disorder with frequent chronic courses and a high lifetime mortality (Arcelus et al., 2011; Steinhausen, 2002). It typically develops during adolescence, with incidence peaking at approximately 15–19 years (Favaro et al., 2019; Grilo & Udo, 2021). Despite severe emaciation and endocrine signals stimulating hunger, individuals with anorexia nervosa (AN) persist in severely limiting their caloric intake. Some of them report that they delay eating a favorite food until the evening or allow themselves to have their favorite food only once a week. This exceptional amount of self-control is often considered a key characteristic of AN (Fairburn et al., 2003; Polivy & Herman, 2002) and plays an important role in emergence, maintenance and treatment resistance of the disorder (Abbate-Daga et al., 2013; Steinhausen, 2002). Maladaptive overcontrol is also apparent in other disorder-related (e.g. excessive physical activity despite physical weakness) and disorder-unrelated behaviors (e.g. perfectionism and high educational achievement: Dalsgaard et al., 2020) as well as in an almost ascetic denial of primary needs and comforts other than food (e.g. pleasant touch: Teaford et al., 2021).

An imbalance between ventral limbic and dorsal executive brain networks has been proposed as a possible underlying mechanism of these behavioral patterns (Kaye et al., 2013). The former enhances the motivational drive (e.g. to eat), the latter inhibition and cognitive control. An imbalance between these networks may explain how impulses to eat are overridden by top-down cognitive control (Brooks, Rask-Andersen, et al., 2012). Both acutely ill and weight-recovered AN have shown altered neural activity in the fronto-parietal control-network across a range of tasks with both disorder-irrelevant (Kaye et al., 2013; Wierenga et al., 2014) and disorder-relevant stimuli (Lloyd & Steinglass, 2018; Simon et al., 2019). Additionally, increased functional connectivity within the fronto-parietal control network has been reported (Boehm et al., 2014; Cowdrey et al., 2014; Ehrlich et al., 2015). Within the fronto-parietal network, the dorsolateral prefrontal cortex (dlPFC) is one of the brain regions for which differential activity has been repeatedly described in AN. It plays a key role in cognitive as well as emotional processing (Dosenbach et al., 2008; Kohn et al., 2014). Increased dlPFC activation in AN has been observed during delay discounting (Decker et al., 2015; Wierenga et al., 2014), set shifting (Zastrow et al., 2009), reward anticipation (Ehrlich et al., 2015) and the viewing of negative emotional (Seidel, et al., 2018) as well as body stimuli (Simon et al., 2019)). DlPFC hyperactivation in AN was also observed in studies focusing on the explicit processing of visual food stimuli (instruction to view or think about eating the food shown in the images: Brooks et al., 2011; Brooks, O'Daly, et al., 2012). Moreover, processing of food stimuli in AN typically evoked increased activation of brain areas associated with bottom-up emotional and/or visual processing in regions of the so-called ‘food-network’ including e.g. the insula, amygdala and fusiform gyrus (Simon et al., 2019; van der Laan et al., 2011). These findings may be interpreted as increased attention to and heightened attribution of salience to food stimuli in AN (Lloyd & Steinglass, 2018), whereas the dlPFC findings indicate an increased involvement of frontal, top-down control mechanisms.

Taken together, there is reliable evidence for increased dlPFC activity in AN evoked by explicit processing of disorder-irrelevant and disorder-relevant stimuli. However, less is known about the neural mechanisms involved in their implicit processing. Especially food cues are prevalent in public places, and they potentially influence food intake to a higher degree than conscious intentions (Elliston et al., 2017). This implies a frequent need for implicit regulation of such stimuli in everyday life. Behavioral data from acute AN point towards a strong implicit representation of negative affect towards high-calorie foods (Spring & Bulik, 2014). In healthy individuals, differential responses to implicit versus explicit processing of emotional stimuli occur in several potentially relevant brain regions (e.g. amygdala: Habel et al., 2007; fusiform gyrus: Monroe et al., 2013). Against this background, the first aim of the current study was to investigate neural activity in the food-network and the dlPFC during performance of a working memory 2-back task including visual food distractor stimuli in individuals with AN. Our task followed previous n-back adaptations with emotional stimuli (e.g. Ladouceur et al., 2009) and included images of high caloric food along with visually similar, emotionally neutral control distractors.

Furthermore, over the last decade, it has become increasingly clear that brain regions should not be considered in isolation, but are part of one or more functional networks (Pessoa, 2014). Therefore, the second aim of our study was to test functional connectivity of the dlPFC and brain regions involved in the bottom-up processing of visual food stimuli. In healthy individuals, an often replicated finding is the top-down regulation of emotionally salient negative stimuli by means of connectivity between dlPFC and amygdala (e.g. Banks et al. (2007)). An altered interplay between dlPFC and amygdala seems to underlie maladaptive emotion processing and regulation across a range of psychopathologies (Sindermann et al., 2021). By means of a clustering analysis of functional connectivity data, Sanders et al. (2015) provided initial evidence for altered network connectivity between top-down and bottom-up food stimulus processing areas in AN. However, the interplay between dlPFC and amygdala during processing of disorder-relevant (e.g. food) stimuli in AN has yet to be investigated.

If the hypothesis of excessive top-down control in individuals with AN is true, one might expect increased dlPFC activity as well as more pronounced negative connectivity between dlPFC and amygdala as a reflection of down-regulation of salient disorder-relevant stimuli in AN. An alternative hypothesis would be that the experience of food stimuli is overwhelming and anxiety provoking in those with AN and might thus disrupt connectivity patterns between regions of the fronto-parietal control network and sub-cortical regions.

## Methods

### Participants

Our initial sample consisted of *n* = 35 adolescent girls and young women diagnosed with anorexia nervosa (AN) and *n* = 62 female healthy controls (HC) within the same age range (12–28 years). Age composition of the sample corresponds well to commonly reported distributions in epidemiological studies (e.g. Favaro et al., 2019). For details on the exclusions during quality control and the selection of the final sample, see supplemental information (SI) 1.1.). Most importantly, we implemented a pair-wise age-matching based on the Munkres algorithm (Munkres, 1957) in order to minimize potentially confounding effects of (neuro-) development and to optimize comparisons. It resulted in a final sample of *n* = 32 AN and *n* = 32 HC with a mean difference of 0.7 years between one pair of participants. AN (mean = 16.26 years, SD = 3.24) and HC (mean = 16.85, SD = 2.97) did not significantly differ with regard to age (see Table 1). Inclusion criteria for AN comprised a body mass index (BMI) below 17.5 kg/m^2^ (if younger than 15.5 years: below the 10th age percentile) and no recent weight gain. All AN were tested within 96 h after admission to a behaviorally oriented nutritional rehabilitation program. HC had to have normal body weight, regular menstruation, and no history of psychiatric disorders. Several exclusion criteria were applied to both groups, including most notably lifetime bulimia nervosa or binge eating pathology, neurological or medical conditions that may influence eating behavior or body weight, and psychotropic medication within 4 weeks prior to the study (see SI 1.1 for details). For all participants, eating-related psychopathology was assessed according to DSM-IV using the Structured Interview for Anorexic and Bulimic Disorders (SIAB-EX: Fichter & Quadflieg, 2001). To supplement the information obtained through the SIAB-EX, we assessed eating disorder-related symptoms (EDI-2: Thiel et al., 1997) and depressive symptoms (BDI-II: Hautzinger et al., 2009) as well as temperament and character traits according to the JTCI (Schmeck et al., 2001). BMI and BMI standard deviation scores corrected for age and gender (BMI-SDS: Kromeyer-Hauschild et al., 2001)) are reported. This study was carried out in accordance with the Declaration of Helsinki and approved by the ethics committee of the Technische Universität Dresden (EK 39,022,012). The privacy rights of the subjects have been observed. All participants (or their legal guardians) gave written informed consent.

**Table 1:** Means (standard deviations) of basic demographic and clinical variables.

	**HC (***n* = **32)**	**AN (***n* = **32)**	**T**	**P**
Age (years)	16.85 (2.97)	16.26 (3.24)	-0.76	.448
IQ	111.41 (7.42)	111.69 (11.76)	0.11	.912
BMI (kg/m^2^)	20.82 (2.39)	14.61 (1.22)	-13.09	<0.001
BMI-SDS	-0.05 (0.75)	-3.28 (1.47)	-11.08	<0.001
EDI-2	151.37 (40.64)	209.10 (41.05)	5.56	<0.001
BDI-II	5.70 (5.53)	21.05 (10.85)	7.13	<0.001
JTCI persistence	47.63 (7.37)	51.44 (8.69)	1.89	.063

*Notes:***BMI** = Body-mass-index, **BMI-SDS** = BMI standard deviation score, **EDI-2** = Eating Disorder Inventory, **BDI-II** = Beck Depression Inventory, **JTCI** = Junior temperament and character inventory. T statistics and p-values are reported for independent sample t-tests between **HC** (healthy controls) and **AN** (individuals with Anorexia Nervosa).

### Task and procedure

**Fig. 1:**
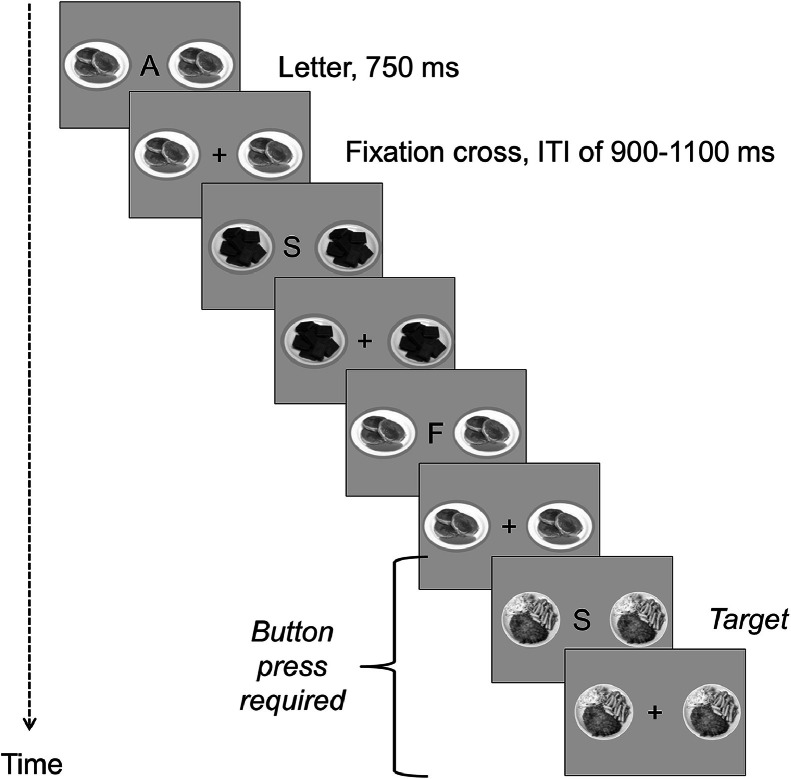
Illustration of the 2-back working memory task.

MRI data were acquired between 8 and 9 AM following an overnight fast to control for acute nutritional intake and diurnal hormone rhythms. During fMRI, participants performed a 2-back working memory task with task-irrelevant food distractor images, which is illustrated in Fig. 1. A sequence of letters (consonants only) was presented centrally (750 ms each), with each letter being followed by a centrally presented fixation cross (inter-trial intervals (ITI): randomly chosen between 900–1100 ms). Each sequence of one letter and one fixation cross was flanked by a new set of two identical distractors, which were either high caloric food or non-food images (depending on the experimental block, design described below). Participants were instructed to attend to the letters only and to press a button with their dominant index finger as quickly as possible whenever a target occurred, i.e. the current letter was identical to the letter presented two trials before (e.g. target in the example of Fig. 1: S-F-S). Food distractors were specifically generated for this experiment; non-food stimuli were scrambled and hence unrecognizable versions of the food images. For detailed information about the stimulus material, see SI 1.2.

For improved suitability for connectivity analyses, we presented high caloric food stimuli and non-food control stimuli in a block design. The task consisted of 3 blocks per distractor category (high caloric food and non-food images). The resulting 6 blocks were presented in pseudo-randomized order. Every block included 30 trials of which 10 were targets. Including short breaks between the blocks, the task took around 7 min to complete. Prior to the MRI scan, participants received detailed instructions and engaged in two training blocks outside the MRI scanner and a third inside the scanner right before the start of the main experiment. Distractors in the training blocks were images of neutral, non-emotional content (e.g. household objects). During the first training block, participants received feedback about the correctness of their responses. To ensure adequate data quality, an accuracy rate of more than 75 % percent during the first two training blocks was required for participation in the main experiment. This led to the exclusion of two AN and three HC prior to analysis. Prior to any analysis, we additionally checked for an adequate behavioral task performance in the main experiment (overall task accuracy >75 %), which was achieved by all participants. QC of fMRI data caused exclusion of one additional AN participant; for fMRI QC procedure, see 2.3.

### Data acquisition and preprocessing

#### Acquisition of MRI data

Images were acquired on a 3T MRI scanner (Trio; Siemens, Erlangen, Germany) equipped with a standard 12 channel head coil. The functional images were recorded with a gradient-echo T2*-weighted echo planar imaging (EPI) sequence with the following parameters: number of volumes = 180–182, number of slices = 42, repetition time = 2410ms. T1-weighted structural brain scans were acquired with a rapid acquisition gradient echo (MP-RAGE) sequence with the following parameters: number of slices = 176, repetition time = 1900ms, slice thickness = 1mm. For more details, see SI 1.3.

#### Preprocessing of MRI data

The functional data were processed in SPM 12 (https://www.fil.ion.ucl.ac.uk/spm/http://www.fil.ion.ucl.ac.uk/spm/) within the Nipype framework (Gorgolewski et al., 2011)https://nipype.readthedocs.io/en/latest/. Realign4D was used to correct the functional images for head motion and staggered slice acquisition; the algorithm allows for an improved reduction of spatio-temporal distortion by simultaneously accounting for slice-timing as well as realignment (Roche, 2011). The functional images were co-registered to the participant's structural T1-image. Subsequently, the functional volumes were normalized to MNI space by means of a group template created in DARTEL (Ashburner, 2007). It included the structural images of all AN or HC, respectively, thus providing a reference unbiased by potential group differences. Finally, an isotropic Gaussian kernel with 8-mm full-width at half-maximum was applied for smoothing.

Quality control was carried out by means of visual inspection and artefact detection tools (ART, www.nitrc.org/projects/artifact_detect/). Additionally, all functional images with motion outliers (>2mm in any direction) and intensity outliers (> 3 SD above the mean of the time series) were discarded. This concerned a maximum of 6.7 % of a single participant's frames (mean: 1.88 %, SD: 1.78 %). Outlier frequency did not differ between AN and HC (intensity: t(62) = 0.139, *p* = .890; motion: t(62) = 0.980, *p* = .331).

#### Additional preprocessing of connectivity data

Connectivity data were analyzed by means of the functional connectivity toolbox *Conn* (version 19.c: Whitfield-Gabrieli & Nieto-Castanon, 2012). A scrubbing procedure was implemented to remove all functional scans with a framewise displacement of >0.9 mm and global signal z-value of >5 SD, which led to the exclusion of mean = 9.1 ± 11.3 frames per participant. In order to validate our results, all analyses were repeated after more conservative scrubbing (>0.5 mm and >3 SD – exclusion of mean = 18.9 ± 16.4 frames/participant). As there were no qualitative differences between the results, we report the findings based on the broader data base. A paired t-test confirmed that the number of valid scans did not differ between AN and HC (t(62) = 0.67, p = .51). The CompCor method (Behzadi et al., 2007) was applied to regress physiological noise and motion parameters from the functional images’ time series, including five nuisance components from white matter and cerebrospinal fluid, respectively. Motion confounds included three translational and three rotational head motion parameters, their first order temporal derivatives, and quadratic effect of all motion confounds (total of 24 parameters). Lastly, data were temporally band-pass filtered (high pass filter: 0.008 - Inf Hz).

### Analysis

#### Behavioral and clinical data

Analyses of behavioral performance, clinical data, questionnaires and their association with extracted fMRI variables were performed in SPSS (Version 27.0, IBM Corp., Armonk, NY, USA). Analyses of performance encompassed average reaction times of trials with correct button presses, error rates (percentage of incorrect responses) as well as commission errors (false positive responses) and omission errors (false negative responses). Reaction times more than 2 standard deviations below and above the mean were excluded from all behavioral analyses (<160 and >1138 ms: 1.3 % of all trials). A univariate mixed-design ANOVA was conducted for each of the four performance measures, including the between-subject factor group (AN versus HC) and the within-subjects factor condition (food versus non-food). Reports of ANOVAs encompassed Greenhouse-Geisser corrected F-statistics (Greenhouse & Geisser, 1959) and partial eta squared (η^2^) as measures of effect size.

#### Neural activity

For individual participants’ preprocessed functional MRI data, a general linear model was fit to each voxel's hemodynamic response in the two experimental conditions: food and non-food distractors. We modelled the presentation of the blocks as a boxcar function with a duration of 52.5 s. Additionally, the six realignment parameters and one regressor for each motion or intensity outlier volume were included into the analysis as nuisance regressors. On the level of group analysis, a linear mixed model was estimated using SPM 12. This model included a binary within-subject variable (condition: food, non-food) and a binary between-subject variable (group: AN, HC). Contrasts were set up to explore the main effect of group and condition as well as their interaction. Age was included as a covariate in all analyses.

As regions of interest (ROI)s, we identified four regions which are involved in bottom-up emotional and/or visual processing of food stimuli (van der Laan et al., 2011) and which have shown differential activation during paradigms with food stimuli in AN: insula (Gizewski et al., 2010), amygdala (Joos et al., 2011), fusiform gyrus (Boehm et al., 2018), and occipital gyrus (Santel et al., 2006). Additionally, we included the dorsolateral prefrontal cortex (dlPFC), which plays a key role in top-down cognitive and affective control (Dosenbach et al., 2008; Kohn et al., 2014). Information on ROI mask creation is provided in SI 1.3. Correction for multiple comparisons was accomplished via small volume correction performed in 3DClustSim (AFNI version 20.1.01, April 2020) including an additional Bonferroni correction for the five ROIs. For details on small volume correction, see SI 1.3. For use in correlational analyses (paragraph 3.5), parameter estimates (beta values) of clusters with significant group differences were extracted using the MarsBar toolbox (Brett et al., 2002). Mean parameter estimates were extracted across all blocks of the task because significant group differences did not vary by condition. For each group, we computed Pearsons correlations between dlPFC parameter estimates and clinical variables (BMI-SDS, EDI-2, BDI-II) as well as the JTCI subscale ‘persistence’; the latter represents a character trait that is strongly related to self-control (Moreira et al., 2012). Exploratory FWE-corrected whole brain analyses are presented in SI 2.3.

#### Connectivity Measures

Hypothesis-driven ROI-to-ROI-analyses were performed to explore connectivity of the left dlPFC (seed ROI) with the left and right amygdala. The seed ROI was defined by the left dlPFC cluster that showed a significant group difference in the preceding analyses (see section 3.3). Target ROIs were defined according to the Harvard-Oxford subcortical structural atlas. Individual ROI-based connectivity maps were computed as Fisher-transformed bivariate correlation coefficients between each seed ROI BOLD time series and each target ROI BOLD time series. Second level comparisons included independent sample t-tests of the correlation coefficients from the AN and HC groups. Correction for multiple testing was achieved by means of threshold-free cluster enhancement (TFCE: Smith & Nichols, 2009) as implemented in the *Conn* toolbox, resulting in a connection-level family-wise error (FWE) correction at *p* < .025 (Bonferroni corrected for the two comparisons). Group comparisons were run across all blocks of the task because the significant group differences of neural activity in the seed ROI did not vary by condition – for further details, see SI Fig. 1.

## Results

### Demographic and clinical data

As shown in Table 1, individuals with AN had a significantly lower BMI, higher eating disorder (EDI-2) and depressive (BDI-II) symptoms when compared with HC. There was a trend towards significantly higher JTCI persistence values for AN compared with HC. AN and HC did not differ with regard to age and IQ.

### Behavioral data

Means (and standard deviations) of the behavioral parameters are displayed in Table 2. Reaction time analyses yielded a significant main effect of condition (F(1,62) = 14.190, *p* < .001, η2 = 0.186), indicating faster responses in the non-food compared with the food condition. There was no significant group difference in reaction times (F(1,62) = 2.953, p = .091, η2 = 0.045) nor a significant interaction between condition and group (F(1,62) = 0.005, p = .943, η2 <0.001). Neither analysis of overall error rates nor of commission and omission errors showed a significant main effect of group, condition or their interaction (all Fs(1,62)≤3.38, all ps≥.07, all η2≤.052 – for detailed error rate results, see SI 2.1). All results remained unchanged when repeating analyses with age as a covariate; see SI 2.2.

**Table 2:** Means (standard deviations) of reaction times, error rates, commission errors and omission errors.

	**AN (Anorexia nervosa)**		**HC (Healthy controls)**	
**Distractor category**	High caloric food	Non-food	High caloric food	Non-food
Reaction time in ms	613.5 (130.2)	588.9 (118.8)	663.9 (114.1)	638.3 (112.9)
Error rate (% of errors in all trials)	6.7 (5.1)	6.1 (6.0)	6.4 (5.0)	5.6 (4.4)
Commission errors (% of errors in trials without a target)	3.3 (2.6)	3.8 (4.2)	3.6 (3.8)	3.2 (4.9)
Omission errors (% of errors in trails with a target)	13.5 (12.4)	10.6 (11.8)	11.9 (9.8)	10.5 (8.4)

*Notes:* (Overall) ‘error rate’ includes commission as well as omission errors. Commission errors are false alarm in no-target trials (two thirds of all trials); omission errors are missing responses in trials with a target (one third of all trials). All error statistics are reported as percentages.

### fMRI data

#### Neural activity

Presenting a successful task manipulation check, contrasts of the food with the non-food condition yielded expected activation in areas associated with the food network, i.e. bilateral fusiform gyri, bilateral occipital gyri and bilateral amygdalae (van der Laan et al., 2011) in both groups. The task manipulation check was further validated by an exploratory whole brain analyses (see SI Table 1). Compared to HC, AN showed clusters of generally increased activation in the left dlPFC and the left occipital gyrus - see Table 3. The same contrast also yielded a supra-threshold cluster in the right dlPFC (MNI coordinates: 28 38 22, cluster size k = 41) which did not remain significant after additional Bonferroni-correction for five ROIs. However, there were no significant interactions between group and condition in any of the ROIs.

**Table 3:** Results of ROI analyses.

Contrast	**ROI**	**H**	**XYZ**	**K**	**Zmax**
Main effects of group					
AN > HC	Occipital gyrus	L	−42 -90 0	198	4.94
	Dorsolateral prefrontal cortex	L	−40 34 28	105	4.09
AN < HC	-	-	-	-	-
Main effects of conditions					
Food > Scrambled	Fusiform gyrus	L	−30 -52 -14	1667	>9.0
		R	32 -46 -16	1692	>9.0
	Occipital gyrus	L	−42 -72 -10	3246	>9.0
		R	44 -78 6	2238	>9.0
	Amygdala	R	32 0 -18	172	5.62
Food < Scrambled	-	-	-	-	-
Interaction group *condition	-	-	-	-	-

*Notes:***ROI =** region of interest, **H** = hemisphere, **XYZ**= MNI coordinates, **K** = cluster size (number of voxels), **Zmax** = peak z-value. **HC** = healthy controls, **AN** = individuals with Anorexia nervosa. All clusters survived small volume correction at *p* < .01, with a voxel-wise threshold of *p* < .001 and ROI-specific cluster extent thresholds (left occipital gyrus *k* = 91, right occipital gyrus *k* = 77, left fusiform gyrus *k* = 50, right fusiform gyrus *k* = 46, amygdala *k* = 12, insula *k* = 62, left dlPFC *k* = 94, right dlPFC *k* = 85).

#### Connectivity measures

**Fig. 2:**
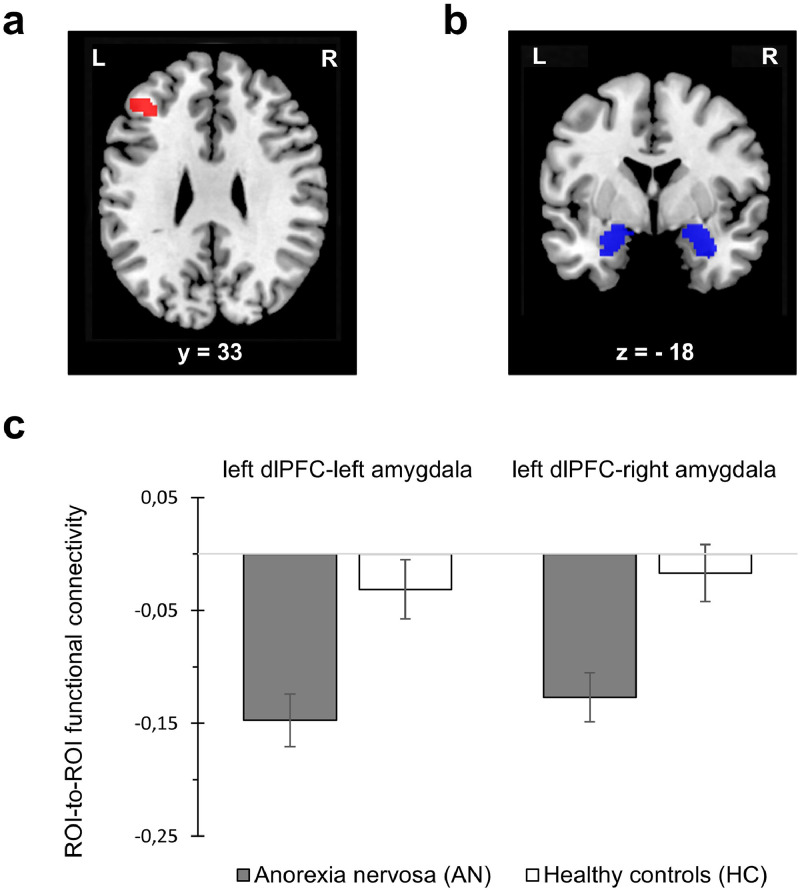
Results of ROI-to-ROI-connectivity analyses. a) Seed ROI: cluster in the left dlPFC that showed significant group differences (AN > HC) across conditions. b) Target ROIs: left and right amygdalae according to the Harvard-Oxford subcortical structural atlas. c) ROI-to-ROI functional connectivity measures (derived from z-transformed bivariate correlation matrices) showed significantly more negative values in AN versus HC for the association between left dlPFC and left amygdala (t(62) = 3.32, *p* = .005) as well as right amygdala (t(62) = 3.30, *p* = .005); these results did not vary by condition (dlPFC-left amygdala: t(62)=1.62, *p* = .222; dlPFC-right amygdala: t(62) = 1.18, *p* = .244). Connectivity tests are FWE-corrected for multiple testing (α = 0.025). Error bars represent standard errors of the mean. dlPFC=dorsolateral prefrontal cortex.

Analyses yielded significant group differences in the connectivity between the identified seed cluster in the left dlPFC and the left as well as the right amygdala, indicating more negative connectivity for AN compared to HC (see Fig. 2). For a condition-wise presentation of these results, see SI Fig. 1.

### Associations between fMRI findings and clinical data

Associations between extracted beta values of fMRI findings with significant group differences and clinical as well as questionnaire variables are presented in SI Table 2. Neither the mean left dlPFC activity nor the functional connectivity measures of left dlPFC were significantly correlated with BMI-SDS, BDI-II, or EDI-2 in any of the two groups. The same was true for the JTCI subscale ‘persistence’.

## Discussion

In this study, fMRI was applied to test dlPFC activity and connectivity during performance of a working memory 2-back task and the implicit processing of visual food distractors in a sample of acutely ill adolescent girls and young women with AN. First, we explored whether increased dlPFC activation would occur selectively during implicit processing of food stimuli in AN. However, the dlPFC activity was generally elevated in AN across conditions. Subsequently, we explored functional connectivity between the dlPFC and the amygdala. We found that the AN group also showed generalized increased negative connectivity between these regions. Against the background of a successful task manipulation check of behavioral and fMRI data and no group differences in task performance, we interpret this pattern of results as indicative of a generalized cognitive top-down control in AN. In the following, we discuss possible explanations and implications.

Negative dlPFC-amygdala connectivity is a well-established neural mechanism of emotion regulation (ER; Berboth & Morawetz, 2021). In our data, this negative connectivity was stronger in AN compared to HC (specifically between the left dlPFC and bilateral amygdalae) during both task conditions. This observation contrasts the potential breakdown of emotion regulation and disruption of dlPFC-amygdala connectivity in other psychiatric disorders, e.g. depression (Dannlowski et al., 2009; Erk et al., 2010). Our finding suggests overregulation in AN, and it is generally in line with that of Seidel et al. (2018), who reported enhanced dlPFC activity and successful down-regulation of amygdala activation in AN during the volitional distancing from disorder-irrelevant, emotionally negative stimuli. Such seemingly intact (or magnified) down-regulation of amygdala responses may explain why some other AN studies on the passive viewing of food stimuli (Holsen et al., 2012; Kerr et al., 2017) including a meta-analysis (Bronleigh et al., 2022) have not been able to replicate an earlier finding of amygdala hyperactivity (Joos et al., 2011). However, in an ER reinterpretation task with disorder-irrelevant stimuli, Steward et al. (2020) found reduced dlPFC activity as well as reduced functional connectivity between dlPFC and amygdala for AN. This finding might differ from ours due to the different kind of stimuli, the explicit instructions and/or the sample's longer average duration of illness.

It is important to emphasize that the observed increased dlPFC activity and negative dlPFC-amygdala connectivity in AN occurred across both experimental conditions: during presentation of food and non-food distractors. Against the background of successful task manipulation checks and no group differences in task performance, this unspecific occurrence seems to reflect a generalized increase in cognitive and affective top-down control in AN. However, given the high emotional salience of food cues in AN (Murray & Strigo, 2018) it might be that they recruit control mechanisms that are sustained for a longer duration of time. We therefore cannot rule out that the aberrant activation and connectivity patterns in the non-food distractor condition represent a carry-over effect.

Our findings seem to also converge with previous reports of increased resting state connectivity of the dlPFC (Cowdrey et al., 2014) and the fronto-parietal control network (Boehm et al., 2018) suggesting that excessive control in AN may occur without any external trigger. Taken together with these resting state findings, the current observation of a condition-unspecific increase in dlPFC activity and connectivity in AN is in line with the assertion that cognitive-affective overcontrol in the disorder may constitute a continuous and sustained process (King et al., 2016; Wierenga et al., 2014). Within the *dual mechanisms framework* of Braver (2012), *sustained* (or *pro-active*) control is described as a control mode in which goal-relevant resources are maintained in a preemptive manner. Qualitatively distinct, *transient* (or *re-active)* control can be understood as a ‘late correction’ mechanism which only mobilizes goal-relevant resources as needed. According to this model, successful self-regulation depends on the right balance between these two modes of control. Braver postulates that they differ in the way they recruit the lateral PFC and presents corresponding fMRI evidence from a number of clinical studies which may reflect an increase in the use of re-active control at the expense of pro-active control (e.g. depressive symptoms: West et al., 2010)). Our findings seem to suggest that AN, in contrast, is characterized by an excess of pro-active control, which may come at the expense of re-active control – which is needed to flexibly adjust processing priorities in accordance with changing demands (King et al., 2019). Future tests of cognitive and ER flexibility (Dann et al., 2023; Miles et al., 2020) using dedicated paradigms would be important to explore this hypothesis.

It is also noteworthy that individuals with AN presented generally increased activity in the left occipital gyrus when compared to HC. This is likely an indicator of increased visual salience (Bradley et al., 2003; Moratti et al., 2004), which would be in line with AN showing an unspecific attentional bias towards all experimental stimuli. We speculate that this may be a consequence of sustained top-down control in AN: attentional resources seem to be deployed to detect potentially goal-relevant events in a preemptive manner. With regard to AN literature, this hypothesis is also compatible with the idea that excessive self-control in AN may be a “habit” (Compan et al., 2015; Walsh, 2013). Such “habitual” self-control may explain why AN show increased dlPFC activation during the anticipation phase of a monetary reward paradigm (Ehrlich et al., 2015) and increased dlPFC activation in response to negative images when instructed not to exert ER (passive viewing condition of Seidel et al., 2018).

Other potential costs of maintaining such elevated levels of control may, especially in combination with a high cognitive rigidity in AN (Miles et al., 2020), include a reduced ability to flexibly allocate control resources in response to changing tasks demands. Support for this hypothesis comes from recent ecological momentary assessments (EMA) of self-control in AN (Fürtjes et al., 2022). In the bigger picture, inflexibility and inherent costliness of control strategies could also help to explain why individuals with AN sometimes show successful ER (Seidel, King, Ritschel, Boehm, Geisler, Bernardoni, Beck, et al., 2018), whereas other paradigms elicited performance deficits and dlPFC hypoactivity (Steward et al., 2020). Increased dlPFC activity in AN may result in a time-delayed “re-bound” of amygdala activation during explicit ER (Pauligk et al., 2021). Also, Seidel, King, Ritschel, Boehm, Geisler, Bernardoni, Holzapfel, et al. (2018) established a link between the successful down-regulation of positive emotions and poorer affective well-being as well as treatment outcome. Together, these data are in line with the notion that negative affect is associated with the exertion of high self-control in AN (Seidel, King, Ritschel, Boehm, Geisler, Bernardoni, Holzapfel, et al., 2018) and emphasize that overcontrol might be resource depleting.

Our results have to be considered in the light of some important limitations. Due to the lack of a control condition for our 2-back working memory task, we cannot exclude that the observed group difference in dlPFC activation may have been influenced by altered 2-back task processing in AN. The same applies to errors and post error-adjustments which were (infrequent but) not modelled given the block design of this fMRI experiment (Ullsperger et al., 2014). However, our behavioral analyses showed comparable working memory task performance of AN and HC (no group-specific main effects or interactions), an observation that is coherent with the absence of group effects in some fMRI working memory studies (e.g. Lao-Kaim et al. (2014)). Second, behavioral and fMRI data yielded the expected effects for food versus non-food distractors in both groups, suggesting that this emotion manipulation worked as intended. Furthermore, the fairly broad age range of our sample (12–29 years) may, despite our strict, pair-wise age-matching and the inclusion of age as a covariate in all analyses, have biased our results due to the influence of neurodevelopmental factors. We have focused on a comparatively young sample with a relatively short duration of illness; the samples age range covered the typical peak incidence. The reported effects might differ in adult-only and/or chronic AN samples.

Taken together, enhanced dlPFC activity and increased dlPFC-amygdala connectivity across all conditions of an emotional 2-back task support the hypothesis of sustained elevated top-down control in AN (Wierenga et al., 2014). Our findings emphasize the necessity to address sustained elevated cognitive and affective top-down control in the treatment of AN (Isaksson et al., 2021). This seems particularly important given the relevance of improved emotion processing for treatment outcome (Oldershaw et al., 2018). A starting point may be the targeting of maladaptive overcontrol via the facilitation of metacognitive awareness - for instance through radically open-dialectical behavior therapy, which has been successfully tested in the treatment of individuals with AN (Lynch et al., 2015). Moreover, neuromodulation of the dlPFC (Dalton et al., 2020) may be considered as viable treatment options, especially as it has been shown to enhance flexibility of food choices in severe cases of AN.

## Declaration of competing interest

The authors declare the following financial interests/personal relationships which may be considered as potential competing interests: Veit Roessner reports a relationship with Lilly that includes: consulting or advisory and speaking and lecture fees. Veit Roessner reports a relationship with Novartis that includes: consulting or advisory, funding grants, and speaking and lecture fees. Veit Roessner reports a relationship with Shire Pharmaceuticals that includes: consulting or advisory, funding grants, and speaking and lecture fees. Veit Roessner reports a relationship with Medice Pharma that includes: speaking and lecture fees. Veit Roessner reports a relationship with Otsuka Companies that includes: funding grants. These funding sources were not involved in the design of the study, the collection and analysis of data, or the decision to publish. S. Pauligk, M. Seidel, R. Ritschel, D. Geisler, A. Doose, I. Boehm, I. Hellerhoff, F. Ludwicki, J. A. King and S. Ehrlich declare no competing interests. V. Roessners declarations of interest are covered in the section "other support". If there are other authors, they declare that they have no known competing financial interests or personal relationships that could have appeared to influence the work reported in this paper.

## Appendix Supplementary materials

Image, application 1
