# Supplementary material for: Overcontrol in anorexia nervosa: Elevated prefrontal activity and amygdala connectivity in a working memory task with food distractors
Source: Int J Clin Health Psychol. 2025 Jan 19;25(1):100544. doi: 10.1016/j.ijchp.2025.100544 (PMC11787555; doi:10.1016/j.ijchp.2025.100544)
Supplement: Supplementary file 1 [file mmc1.docx]

Supplementary Information­­­­­ for “Overcontrol in anorexia nervosa: elevated prefrontal activity and amygdala connectivity in a working memory task with food distractors**”***­­*

[1. Methods 2](#_Toc176037916)

[1.1. Participants 2](#_Toc176037917)

[1.2. Stimuli 3](#_Toc176037918)

[1.3. Data acquisition and analysis 3](#_Toc176037919)

[2. Results 4](#_Toc176037920)

[2.1. Detailed results of error rate analyses 4](#_Toc176037921)

[2.2. Behavioral analyses including age as a covariate 4](#_Toc176037922)

[2.3. fMRI data: exploratory whole-brain analysis 5](#_Toc176037923)

[2.4. Connectivity measures 6](#_Toc176037924)

[2.5. Associations between fMRI findings and clinical data 6](#_Toc176037925)

[3. References 7](#_Toc176037926)

## **Methods**

## **Participants**

Our preliminary sample consisted of 35 females diagnosed with anorexia nervosa (AN) and a total of 62 female healthy controls (HC) within the same age range (12-28 years). HC participants were oversampled due the requirements of another study focused on adults. In order to minimize potentially confounding effects of (neuro-) development and to optimize comparisons with the remaining n=32 predominantly adolescent AN, we implemented a pair-wise case-control matching algorithm, which randomly selected n=32 age-matched HC counterparts from the initial, larger pool of HC (for details, see main manuscript section 2.1).

The recruitment process and all psychological assessments were conducted by clinically experienced research assistants under the supervision of a senior child and adolescent psychiatrist. AN participants were inpatients at a child and adolescent psychiatry or psychosomatic medicine department. They underwent the assessment within 96 hours after admission to a behaviorally oriented nutritional rehabilitation program. In AN, exclusion criteria comprised any history of bulimia nervosa or binge eating, substance abuse, schizophrenia, psychosis or bipolar disorder. Five AN were diagnosed with other comorbid psychiatric disorders (including depressive episodes or dysthymia (n=3), obsessive compulsive disorder (n=1), social phobia (n=1), undifferentiated somatoform disorder (n=1), and avoidant personality disorder (n=1)). Comorbid diagnoses in AN were made by an expert clinician following a careful, interdisciplinary diagnostic procedure during the inpatient treatment. AN were excluded if substantial weight gain was reported in the four weeks preceding the first study point (>2.5 kg).

HC were recruited through public advertisement in schools and universities. HC were excluded if they had a lifetime BMI below 18.5kg/m^2^ or above 30kg/m^2^ (<10th and >97th age percentile, if younger than 18 years). HC had to have a regular menstrual period and no history of psychiatric disorder. Psychiatric diagnoses in HC were assessed by means of a semi-structured research interview. This interview also included questions on further exclusion criteria in both experimental groups, namely psychotropic medication (up to 4 weeks prior to the study) and inflammatory, metabolic, neurological, or other chronic disorders with potential influence on appetite, eating behavior, or body weight (such as diabetes, thyroid disease, organic brain syndrome). Furthermore, participants of both groups were excluded if they were currently pregnant, breast-feeding, or had an intelligence quotient <85. For intelligence diagnostics, we applied the German adaptations’ short version of the Wechsler Adult Intelligence Scale (Donnell et al., 2007) or the Wechsler Intelligence Scale for Children (Waldmann, 2008). Participants were paid for their participation.

## Stimuli

Distractor stimuli were specifically generated for this experiment. The food pictures depict lunch plates (single plates taken from above) which are typical for the region and often consumed by young people. The amount of food per plate corresponded to what is recommended for lunch by the German Society for Nutrition. In a behavioral pilot study, a separate sample of 11 adolescent participants with acute AN was asked to guess the calorie content and rate their anxiety when watching the food stimuli. They were presented with all high caloric stimuli of the study and 6 low caloric control stimuli. Results confirmed that the study material was perceived as high-caloric and fearsome by the participants with acute AN.

Non-food stimuli were scrambled and hence unrecognizable versions of the food pictures. They were generated with GNU Image Manipulation Program (GIMP, Kimball & Mattis, 2012) by dividing the photographs into 625 rectangular fragments each and spatially scrambling the layouts of the mosaics. This procedure ensured a high comparability of the two distractor sets with regard to visual properties: There were no differences in luminance and number of pixels between the two distractor categories (food vs. non-food). Target letters and fixation crosses were presented in black font. All pictures were displayed on a grey background.

## Data acquisition and analysis

**MRI Data Acquisition**. The functional images were recorded with a gradient-echo T2*-weighted EPI sequence (TR=2410 ms; TE=25 ms; flip angle=80°). A total of 180-182 volumes were obtained (42 transversal slices orientated 30° clockwise to the AC-PC line (to reduce signal dropout in the orbitofrontal regions), FOV=192×192 mm, 2 mm slice thickness, 1 mm gap, in-plane resolution of 64×64 pixels=voxel size of 3×3×2mm^3^, bandwidth of 2112 Hz/pixel). Structural brain scans were acquired with a T1-weighted rapid acquisition gradient echo (MP-RAGE) sequence (176 slices, flip angle=9°, TR=1900ms, TE=2.26ms, FOV=256×224mm, 1×1×1mm^3^ voxel size, bandwidth of 200 Hz/pixel). Task presentation and recording of the behavioral responses were carried out via Presentation (Neurobehavioral Systems, Inc., Albany, CA). Stimuli were presented onto a mirror-based back-projection screen attached to the head coil of the scanner. The web-based electronic data capture tool REDCap (Harris et al., 2009) was used to manage data collection.

**Region of Interest (ROI) analyses.** Single anatomical masks which included left and right hemispheric regions were used for ROI analyses of amygdala and insula. Separate masks for the left and right hemisphere were created for all other ROIs. The masks of amygdala, insula and fusiform gyri were derived from the Automated Anatomic Labelling (AAL) atlas provided within the Wake Forest University (WFU) PickAtlas for SPM (Maldjian et al., 2003; Tzourio-Mazoyer et al., 2002). Masks for the occipital gyri and dlPFC were created by merging the corresponding AAL regions within the WFU PickAtlas (occipital gyrus: inferior, middle and superior occipital gyrus; dlPFC: superior and middle frontal gyrus). In both dlPFC masks, parts posterior to y=24 (MNI space) were excluded as in our previous work (Ehrlich et al., 2010, 2012).

**Small volume correction via 3DClustSIM.** Correction for multiple comparisons was accomplished via small volume correction performed in 3DClustSim (AFNI version 20.1.01, April 2020 - <http://afni.nimh.nih.gov/pub/dist/doc/program_help/3dClustSim.html>). Based on Monte Carlo Simulations, this program estimates the cluster size at which the chance of false positives falls below a given alpha level (bonferroni-corrected for 5 ROIs: α=.01, two-sided) for a given voxelwise p-value level (p=.001). Separate simulations were run for each ROI. A cluster was considered significant if it exceeded the following minimum cluster sizes: left occipital gyrus k =91, right occipital gyrus k=77, left fusiform gyrus k=50, right fusiform gyrus k=46, amygdala k=12, insula k=62, left dlPFC k=94, right dlPFC k=85.

## Results

## Detailed results of error rate analyses

None of the error rate analyses showed a main effect of group (overall error rate: F(1,62)=0.127, p=.723, η2=.002; commission errors: F(1,62)=0.039, p=.843, η2=.001; omission errors: F(1,62)=0.134, p=.715, η2=.002), main effect of condition (overall error rate: F(1,62)=1.687, p=.199, η2=.026, commission errors: F(1,62)=0.004, p=.947, η2=.000, omission errors; F(1,62)=3.384, p=.071, η2=.052) or interaction between group and condition (overall error rate: F(1,62)=0.004, p=.948, η2=.000; commission errors: F(1,62)=1.270; p=.264, η2=.020, omission errors: F(1,62)=0.453, p=.503, η2=.007).

## Behavioral analyses including age as a covariate

Reaction time analyses yielded a significant main effect of condition (F(1,61)=10.028, p=.002, η2=.141), indicating faster responses in the non-food compared with the food condition. There was no significant group difference in reaction times (F(1,61)=2.774, p=.101, η2=.043) nor a significant interaction between condition and group (F(1,61)=0.028, p=.869, η2 <.001). None of the error rate analyses showed a main effect of group (overall error rate: F(1,61)=0.980, p=.326, η2=.016; commission errors: F(1,61)=0.017, p=.896, η2<.001; omission errors: F(1,61)=0.753, p=.389, η2=.012), main effect of condition (overall error rate: F(1,61)=0.042, p=.838, η2=.001, commission errors: F(1,61)=0.004, p=.949, η2<.001, omission errors; F(1,61)=0.058, p=.811, η2=.001) or interaction between group and condition (overall error rate: F(1,61)=0.012, p=.915, η2<.001; commission errors: F(1,61)=1.249; p=.268, η2=.020, omission errors: F(1,61)=0.372, p=.544, η2=.006).

## fMRI data: exploratory whole-brain analysis

Topographical and statistical details of an exploratory whole-brain analysis are provided in **SI Table 2**. When compared to non-food pictures, food pictures elicited increased activity in a network spanning the bilateral fusiform gyri, occipital gyri, posterior orbitofrontal cortices, the right amygdala and the left posterior parietal gyrus. AN showed significantly increased activation within the middle occipital gyrus, the precentral gyrus and the middle frontal gyrus compared with HC. There was no significant interaction between condition and group

**SI Table 1**. Results of the exploratory whole-brain fMRI analysis.

| **Contrast** | **Brain region** | **H** | **XYZ** | **K** | **Zmax** | **P** |
| --- | --- | --- | --- | --- | --- | --- |
| Main effects of group |  |  |  |  |  |  |
| AN > HC | Middle occipital gyrus | L | -42 -90 0 | 13 | 4.94 | 0.01 |
|  | Precentral gyrus | R | 26 -4 48 | 25 | 4.94 | 0.004 |
|  | Middle frontal gyrus | R | 28 38 20 | 1 | 4.68 | 0.038 |
| AN < HC | - | - | - | - | - | - |
| Main effects of condition |  |  |  |  |  |  |
| Food > Scrambled | Fusiform and occipital gyrus | L | -30 -52 -14 | 4450 | >9.0 | <.000 |
|  | Fusiform and occipital gyrus | R | 32 -46 -16 | 3670 | >9.0 | <.000 |
|  | Posterior orbitofrontal cortex | L | -26 30 -14 | 88 | 6.07 | <.000 |
|  | Amygdala | R | 32 0 -18 | 119 | 5.62 | <.000 |
|  | Olfactory cortex | R | -24 8 -14 | 26 | 5.58 | 0.004 |
|  | Temporal pole / middle temporal gyrus | R | 28 4 -36 | 5 | 5.56 | 0.022 |
|  | Posterior orbitofrontal cortex | R | 26 30 -14 | 38 | 5.27 | 0.002 |
|  | Superior parietal gyrus | L | -12 -82 50 | 14 | 4.99 | 0.009 |
|  | Posterior orbitofrontal cortex | R | 38 32 -16 | 14 | 4.98 | 0.009 |
| Food < Scrambled | - | - | - | - | - | - |
| Interaction group * condition | - | - | - | - | - | - |
| *Notes:* **H** = hemisphere, **XYZ**= MNI coordinates, **K**=cluster size (number of voxels), **Zmax** = peak z value, **P**=p-value of cluster, FWE-corrected (α=.05). **AN** = individuals diagnosed with anorexia nervosa, **HC** = healthy controls. | | | | | | |

## Connectivity measures

**SI Figure 1. Results of ROI-to-ROI-connectivity analyses, per condition.** Conditions-wise functional connectivity measures as derived from z-transformed bivariate correlation matrices. Error bars represent standard errors of the mean. Seed ROI: cluster in the left dlPFC that showed significant group differences in the main connectivity analyses, see main manuscript, 3.3. Target ROIs: left and right amygdala according to the Harvard-Oxford subcortical structural atlas. **dlPFC**=dorsolateral prefrontal cortex.

## Associations between fMRI findings and clinical data

| **SI Table 2**. Correlation coefficients r and corresponding values of significance (p) for the associations between extracted fMRI betas values, clinical and questionnaire variables. | | | | | | |
| --- | --- | --- | --- | --- | --- | --- |
|  | **mean activation left dlPFC** | | **connectivity left dlPFC with left amygdala** | | **connectivity left dlPFC with right amygdala** | |
|  | **AN** | **HC** | **AN** | **HC** | **AN** | **HC** |
| **BMI-SDS** | - .136 (.46) | .287 (.11) | - .178 (.33) | .136 (.46) | - .103 (.58) | .076 (.68) |
| **EDI** | - .259 (.16) | .137 (.46) | .064 (.73) | .059 (.75) | - .081 (.66) | - .081 (.66) |
| **BDI** | - .171 (.35) | - .014 (.94) | .015 (.94) | - .084 (.65) | .067 (.72) | - .194 (.29) |
| **JTCI persistence** | - .066 (.72) | .091 (.62) | - .140 (.45) | - .071 (.70) | - .041 (.82) | - .186 (.31) |
| *Notes.* **BMI** = Body-mass-index, **BMI-SDS** = BMI standard deviation score, **EDI** = Eating Disorder Inventory, **BDI** = Beck Depression Inventory, **JTCI** = Junior temperament and character inventory. **HC** = healthy controls, **AN** = individuals diagnosed with Anorexia Nervosa. None of the correlations was significant. | | | | | | |

## **References**

Donnell, A. J., Pliskin, N., Holdnack, J., Axelrod, B., & Randolph, C. (2007). Rapidly-administered short forms of the Wechsler Adult Intelligence Scale—3rd edition. *Archives of Clinical Neuropsychology*, *22*(8), 917–924. https://doi.org/10.1016/j.acn.2007.06.007

Ehrlich, S., Brauns, S., Yendiki, A., Ho, B.-C., Calhoun, V., Schulz, S. C., Gollub, R. L., & Sponheim, S. R. (2012). Associations of Cortical Thickness and Cognition in Patients With Schizophrenia and Healthy Controls. *Schizophrenia Bulletin*, *38*(5), 1050–1062. https://doi.org/10.1093/schbul/sbr018

Ehrlich, S., Morrow, E. M., Roffman, J. L., Wallace, S. R., Naylor, M., Bockholt, H. J., Lundquist, A., Yendiki, A., Ho, B.-C., White, T., Manoach, D. S., Clark, V. P., Calhoun, V. D., Gollub, R. L., & Holt, D. J. (2010). The COMT Val108/158Met polymorphism and medial temporal lobe volumetry in patients with schizophrenia and healthy adults. *NeuroImage*, *53*(3), 992–1000. https://doi.org/10.1016/j.neuroimage.2009.12.046

Harris, P. A., Taylor, R., Thielke, R., Payne, J., Gonzalez, N., & Conde, J. G. (2009). Research Electronic Data Capture (REDCap)—A metadata-driven methodology and workflow process for providing translational research informatics support. *Journal of Biomedical Informatics*, *42*(2), 377–381. https://doi.org/10.1016/j.jbi.2008.08.010

Kimball, S., & Mattis, P. (2012). *Gnu image manipulation program* [Computer software]. The GIMP Development Team.

Maldjian, J. A., Laurienti, P. J., Kraft, R. A., & Burdette, J. H. (2003). An automated method for neuroanatomic and cytoarchitectonic atlas-based interrogation of fMRI data sets. *NeuroImage*, *19*(3), 1233–1239. https://doi.org/10.1016/S1053-8119(03)00169-1

Tzourio-Mazoyer, N., Landeau, B., Papathanassiou, D., Crivello, F., Etard, O., Delcroix, N., Mazoyer, B., & Joliot, M. (2002). Automated Anatomical Labeling of Activations in SPM Using a Macroscopic Anatomical Parcellation of the MNI MRI Single-Subject Brain. *NeuroImage*, *15*(1), 273–289. https://doi.org/10.1006/nimg.2001.0978

Waldmann, H.-C. (2008). Kurzformen des HAWIK-IV: Statistische Bewertung in verschiedenen Anwendungsszenarien. *Diagnostica*, *54*(4), 202–210. https://doi.org/10.1026/0012-1924.54.4.202
